# Dataset on preliminary phytochemical analysis and antioxidant activity of selected invasive alien plant species used in the treatment of sexually transmitted infections in Waterberg district, South Africa

**DOI:** 10.1016/j.dib.2019.104281

**Published:** 2019-07-17

**Authors:** Lesibana Petrus Maema, Martin Johannes Potgieter, Amidou Samie

**Affiliations:** a Department of Biodiversity, School of Molecular and Life Sciences, University of Limpopo, Private Bag X1106, Sovenga 0727, South Africa; b Department of Microbiology, School of Mathematics and Natural Sciences, University of Venda, Private Bag X5050, Thohoyandou, South Africa

**Keywords:** Sexually transmitted infections, Medicinal plants, Phytochemical analysis, Antioxidant activity

## Abstract

The current dataset follows the published article [1]. The dataset provides preliminary phytochemical analysis and antioxidant activity of selected invasive alien plant used by Bapedi Traditional Health Practitioners to treat sexually transmitted infections (STIs). It was evident that seven STIs are treated with herbal remedies of the documented plant species. Informational on the medicinal plant uses and the use categories of sexually transmitted infections are presented on table 1. Table 2 shows the yield of plant extracts. Detailed data on phytochemical analysis and antioxidant activity are presented on Fig 1 and 2 respectively. Rf values of separated compounds are provided in Table 3. The data contains both qualitative and quantitative information.

**Table:** Specifications Table

Subject area	Botany, pharmacology
More specific subject area	Ethnobotany, phytochemistry, antioxidant activity
Type of data	Tables and figures
How data was acquired	Semi-structured questionnaire, guided field survey and experimental design
Data format	Raw and processed
Experimental factors	Plant species were selected based on previous ethnobotanical survey [1]. Plants with high fidelity level were selected for phytochemical analysis and antioxidant activity
Experimental features	Quantitative data analysis of selected plants, plant material collections, extraction of plant materials, phytochemical analysis and antioxidant activity using qualitative analysis.
Data source location	Waterberg District, South Africa (23°10′−24°20′S and 28°10'−29°10′E) for collected plant materials.
Data accessibility	The data is with the article.
Related research article	[1]

**Value of the Data**	

• The datasets provide information on the most frequently used medicinal plants to treat sexually transmitted infections (STIs). • The data presented encourage further antimicrobial studies against the strains that are responsible for STIs. This could validate Bapedi traditional medicine practices, especially regarding the management of STIs. • The data outlined on the figures contain qualitative information on the phytochemical analysis and antioxidant activity which could assist in pinpointing plant extract with promising drug lead. • The combination of plant species is detailed in the published article [1] which could be used as the basis in the synergistic studies for any interactions.	

## 1 Data

**Fig. 1:**
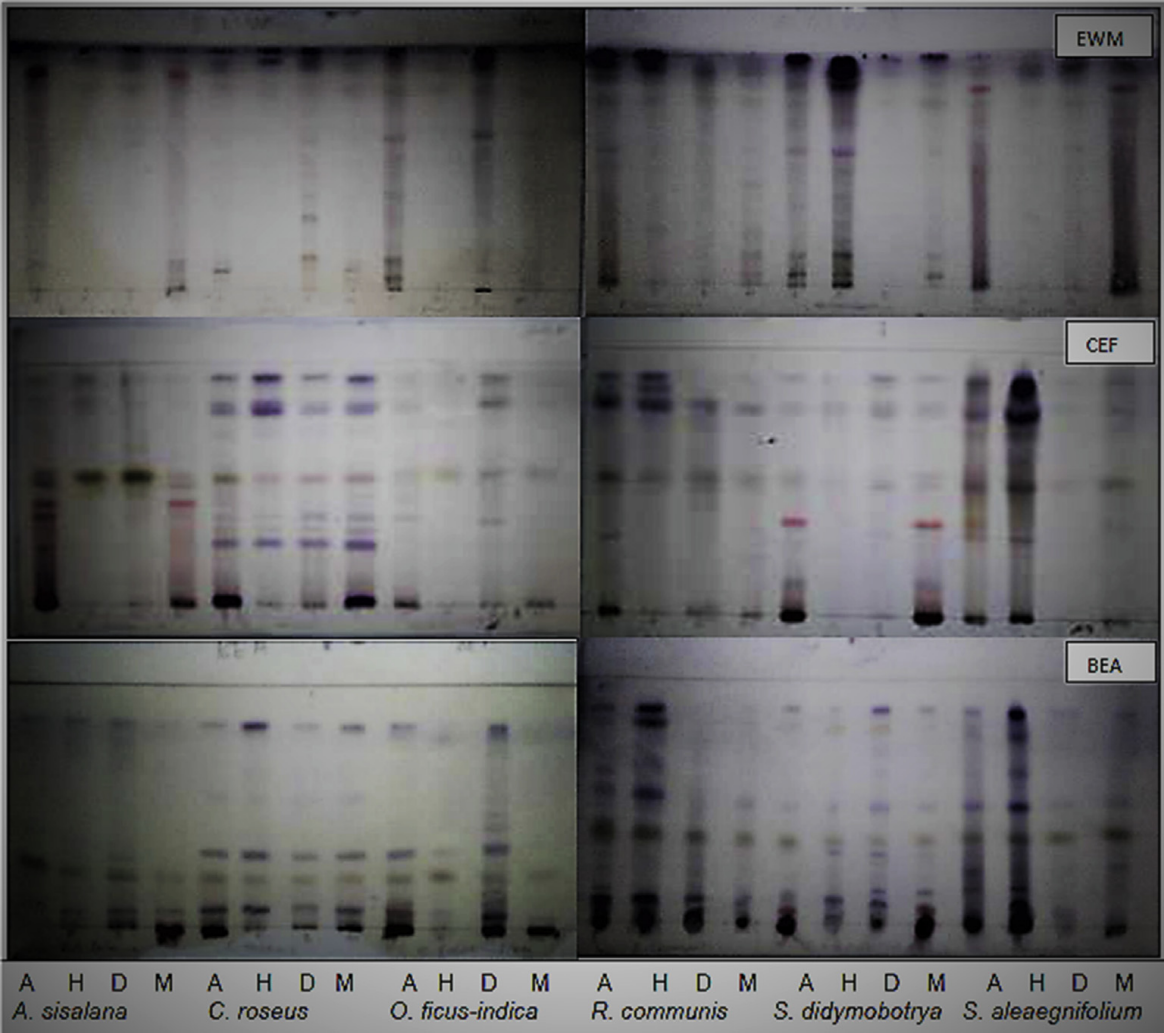
Thin layer chromatography sprayed with vanillin-sulphuric acid showing phytochemical constituents of six plants extracted with four solvents (A: acetone, H: hexane, D: dichloromethane, M: methanol) separated with three solvent systems (BEA, CEF, EMW).

**Fig. 2:**
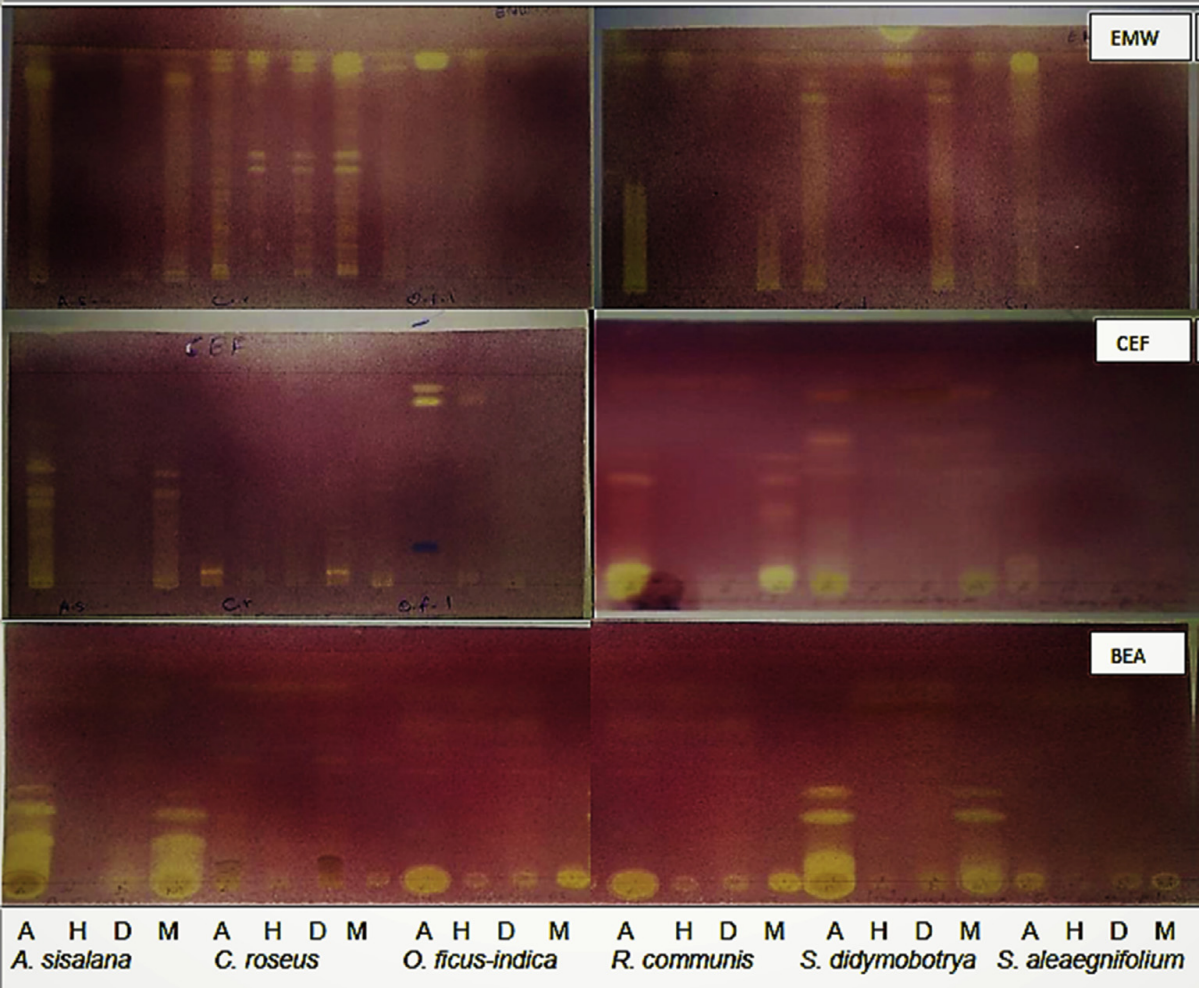
Thin layer chromatography showing antioxidant activity of six plants extracted with four solvents (A: acetone, H: hexane, D: dichloromethane, M: methanol) separated with three solvent systems (BEA, CEF, EMW).

The data shared entails information on medicinal plants used to treat sexually transmitted infection (STIs) (Table 1). The plant materials were extracted using solvents of varying polarities. The yield of crude extracts from roots of selected plant species were calculated as presented on Table 2. Plants were selected because of high number of FL values (Table 1). Moreover, preliminary phytochemical analysis of plant crude extracts was analysed using a thin layer chromatography (TLC) (Fig. 1). The antioxidant activity of plant extracts is portrayed on the TLC plate with a yellow band against purple background (Fig. 2). Retention factor (*R*_*f*_) values for each separated compound were calculated, including compounds with antioxidant activity (Table 3).

**Table 1:** Medicinal plants most frequently used to treat sexually transmitted infections.

Plant species	STIs treated	Frequency of report	Fidelity level
*Catharanthus roseus* (L.) G.Don	Gonorrhoea	11	57.9
	Chlamydia	2	10.5
	Syphilis	3	15.8
	HIV/AIDS	1	5.3
	Genital warts	2	10.5
*Agave sisalana* Perrine ex Engelm.	Gonorrhoea	4	33.3
	Chlamydia	4	33.3
	Syphilis	2	16.7
	*Makgoma*	1	8.3
	Genital warts	1	8.3
*Opuntia ficus-indica* Mill.	Gonorrhoea	7	63.6
	Chlamydia	1	9.1
	Syphilis	1	9.1
	*Makgoma*	1	9.1
	Genital warts	1	9.1
*Ricinus communis* L.	Gonorrhoea	3	33.3
	Chlamydia	2	22.2
	Syphilis	1	11.1
	*Makgoma*	3	33.3
*Senna didymobotrya* (Fresen.) H.S.Irwin & Barneby	Gonorrhoea	3	30.0
	Chlamydia	2	20.0
	Syphilis	1	10.0
	*Mokabe*	3	30.0
	Genital warts	1	10.0
*Solanum elaeagnifolium* Cav.	Gonorrhoea	6	37.5
	Chlamydia	3	18.8
	Syphilis	3	18.8
	HIV/AIDS	1	6.3
	*Makgoma*	3	18.8
	Genital warts	2	12.5

**Table 2:** Percentage yield from powdered roots material of different plant species using different extraction solvents: acetone [A], hexane [H], dichloromethane [D] and methanol [M].

Plant species	% of plant material extracted (mg)				Average
	A	H	D	M	
*Agave sisalana*	3.9	1.3	1.4	9.9	4.1
*Catharanthus roseus*	9.5	1.4	2.5	9.5	5.7
*Ricinus communis*	1.9	1.5	0.4	9.1	3.2
*Opuntia ficus-indica*	0.7	0.3	0.3	0.8	0.5
*Senna didymobotrya*	7.6	0.5	1.2	12.1	5.4
*Solanum elaeagnifolium*	0.9	1.9	0.3	4.3	1.8
Average	4.1	1.2	1	7.6	3.5

**Table 3:** Phytoconstituets profiles and antioxidants of roots extracted with acetone, hexane, dichloromethane and methanol using three solvent systems.

Solvent system	A. sisalana			C. roseus			O. ficus-indica			R. communis			S. didymobotrya			S. eleaegnifolium		
	EMW	CEF	BEA	EMW	CEF	BEA	EMW	CEF	BEA	EMW	CEF	BEA	EMW	CEF	BEA	EMW	CEF	BEA
Acetone	0.12	0.13	0.06	**0.05**	**0.04**	0.03	0.06	0.37	0.03	**0.06**	0.29	0.06	0.03	0.13	**0.09**	0.37	0.28	0.06
	0.51	0.24	0.09	0.09	0.24	0.06	0.09	0.56	0.06	**0.09**	**0.43**	0.09	0.13	0.20	0.13	0.52	0.29	0.09
	0.83	0.29	**0.13**	0.13	0.29	0.24	0.13	0.87	0.09	**0.29**	0.51	0.13	0.51	0.27	**0.27**	0.60	0.37	0.13
	**0.87**	**0.37**	**0.20**	0.62	0.37	0.29	0.34	0.96	0.24	**0.37**	0.77	0.37	0.60	0.37	**0.37**	0.8	0.51	0.20
	0.91	**0.43**	**0.29**	0.87	0.56	0.56	0.62		0.29	0.53	0.83	0.53	0.69	**0.46**	0.53	0.87	0.69	0.24
		**0.51**	**0.37**	0.92	0.83	0.83	0.87		0.37	0.60	0.87	0.60	**0.81**	0.51	0.83	0.96	0.77	0.37
		**0.56**	0.40	**0.95**	0.87		**0.95**		0.73	0.80		0.87	**0.87**	**0.56**	0.87		0.91	0.43
		0.87	0.87		0.96		0.97		0.79	0.91			**0.96**	**0.77**				0.87
		0.97							0.83	**0.96**				0.87				
Hexane	0.73	0.56	0.06	**0.43**	0.24	0.06	0.73	0.56	0.06	0.24	0.51	0.09	0.03	0.51	0.03	0.93	0.28	0.06
	0.91	0.87	0.24	**0.54**	0.29	0.20	0.86	0.92	0.20	0.80	0.77	0.37	0.13	0.77	0.09	**0.96**	0.29	0.09
		0.97	0.37	0.87	0.37	0.29	**0.95**	**0.87**	0.24	0.96	0.83	0.53	0.51	0.83	0.29		0.37	0.24
			0.87	**0.95**	0.56	0.56		**0.93**	0.29		0.87	0.87	0.60	0.91	0.37		0.52	0.37
					0.83	0.83			0.77			0.91	0.69		0.53		0.69	0.87
					0.87								0.81		0.83		0.77	
					**0.96**								0.87		0.87		0.91	
													0.96					
dichloromethane	0.60	0.56	0.06	0.60	0.24	0.21	0.08	0.37	0.06	0.43	0.51	0.06	0.81	0.32	0.09	0.64	0.51	0.09
	0.83	0.87	0.20	0.09	0.29	0.24	0.62	0.56	0.09	0.51	0.77	0.09	**0.87**	0.48	0.13	0.80		0.24
	0.91	0.97	0.24	0.14	0.37	0.34	0.86	**0.87**	0.13	0.80		0.13	**0.96**	0.51	0.24	0.93		0.37
			0.29	0.17	0.56	0.62	0.8	0.96	0.20	0.96		0.25		0.77	0.29	0.96		
			0.83	0.21	0.83	0.87	0.95		0.24			0.29		0.83	0.37			
				0.24	0.87	0.92	0.97		0.37			0.37		0.87	0.53			
				**0.34**	0.91	0.95			0.43			0.91		0.91	0.83			
				**0.54**	0.96				0.47						0.87			
				0.62					0.69									
				0.87					0.77									
				**0.95**					0.83									
Methanol	0.03	0.24	0.06	**0.05**	**0.04**	0.03	0.06	0.56	0.06	**0.03**	0.20	0.04	0.03	0.13	**0.09**	0.37	0.37	0.06
	0.09	0.29	**0.13**	0.09	0.24	0.06	0.73	0.87	0.24	**0.13**	**0.32**	0.06	0.13	0.27	0.13	0.52	0.520.77	0.09
	0.13	0.37	**0.20**	0.10	0.29	0.09	0.87	0.96		**0.24**	**0.43**	0.13	**0.60**	0.37	**0.27**	0.60	0.91	0.20
	**0.87**	**0.43**	**0.29**	0.37	0.37	0.13				0.37	0.51	0.25	**0.81**	**0.46**	**0.37**	0.8		0.24
	0.91	**0.51**	0.87	**043**	0.56	0.15				0.43	**0.54**	0.29	**0.87**	0.51	0.53	0.87		0.37
		**0.56**		**0.51**	0.83	0.20				0.60	0.77	0.37		**0.56**	0.83	0.96		0.87
				**0.54**	0.87	0.29				0.80		0.91		**0.77**	0.87			
				0.87	0.96	0.53				0.87				0.87				
				0.91		0.83				**0.96**								
				**0.95**														

**Note**: bolded *Rf* values are phytoconstituents with antioxidant activity.

## 2 Experimental design, materials, and methods

### 2.1 Ethnobotanical survey and plant extraction

An ethnobotanical survey was conducted as elaborated in the published article [1]. Based on ethnobotanical information provided by traditional health practitioners, plant materials (roots) were collected, dried, and grinded. Separate aliquots of finely ground plant material (5 g) were extracted with 50 ml of solvents of increasing polarities: hexane, dichloromethane, acetone and methanol for at least 72 h with frequent shaking on a shaking incubator. The samples were filtered through Whatman No.1 filter paper and filtrates were used for phytochemical screening. Second extraction procedure was executed, and filtrates were pre-weighed in the glass vials and air-dried under a stream of cold air. The quantity of plant material extracted was determined by comparing the amount of extract with the original plant material. The extracts were stored in airtight glass vials in the dark until used for antioxidant and phytochemical assays. The dry plant extracts were reconstituted into acetone making 100 mg/ml stock solution used for biological assays.

### 2.2 Qualitative phytochemical analysis

Chemical constituents of the extracts were analysed using aluminium-backed Thin Layer Chromatography (TLC) plates (ALIGRAM_SIL g/UV 254-MACHEREY-NAGEL, Merck), that was developed with three eluent systems developed in the botany laboratory (UNIVEN). Ethylacetate: methanol: water: 40:5:0.4 [EMW] (polar) Chloroform: ethylacetate: formic acid: 5:4:1 [CEF] (intermediate polarity) Benzene: ethanol: ammonia hydroxide: 90:10:1 [BEA] (non-polar/basic) [2].

The stock solution (100 mg/ml) of the extracts were re-dissolved to the concentration of 10mg/ml in acetone. Acetone was selected due to its extraction capability. Development of the chromatograms was under eluent-saturated conditions. Approximately 100 μg aliquot (10 mg/ml) was applied on the TLC plates in a 1 cm band and developed without delay to minimize the possibility of photo-oxidative change. The separated components were visualized under visible and UV light (254 and 360 nm, Lamina flow). For the detection of chemical compounds not visible under UV light, vanillin-sulphuric acid reagent (0.1 g vanillin, 28 ml methanol (MeOH); 1 ml sulphuric acid) was sprayed on the chromatogram and heated at 110 °C for colour development.

### 2.3 Antioxidant compounds analysis

The antioxidant compounds of each plant extract were determined by using a qualitative 2, 2-diphenyl-1-picrylhydrazyl (DPPH). This assay is preferred because it is used to provide stable free radicals. A solution of 0.2% DPPH in MeOH was prepared and then sprayed on the plates (until they became wet) and allowed to dry in a fume cupboard. The presence of yellow zones against a purple background on chromatograms indicated the presence of the scavenging activity of free radicals by compounds present in the plant extracts.

### 2.4 Data analysis

Data were captured in Microsoft Excel 2016 programme and were later analysed by descriptive statistics. Quantitative tool such as Fidelity Level (FL), was used to analyse the importance of medicinal plants and informants' knowledge about categories of STIs [3]. Compound bands on the TLC were then used to calculate *R*_*f*_ values with the formula *R*_*f*_ = distance moved by the compounds/distance moved by the solvents front.
