# Supplementary material for: Dataset on preliminary phytochemical analysis and antioxidant activity of selected invasive alien plant species used in the treatment of sexually transmitted infections in Waterberg district, South Africa
Source: Data Brief. 2019 Jul 17;25:104281. doi: 10.1016/j.dib.2019.104281 (PMC6669344; doi:10.1016/j.dib.2019.104281)
Supplement: Multimedia component 1 [file mmc1.docx]

**Lesibana Petrus Maema**

South Africa, University of Limpopo, Department of Biodiversity, Sovenga (0727), | +27 64 777 5225 | plesibana@gmail.com

**20 March 2019**

Scientific Editor

Data in Brief

Declaration of interest

We wish to confirm that there are no known conflicts of interest associated with this publication and there has been no significant financial support for this work that could have influenced its outcome. We confirm that the manuscript has been read and approved by all named authors and that there are no other persons who satisfied the criteria for authorship but are not listed.

We further confirm that the order of authors listed in the manuscript has been approved by all of us. We confirm that we have given due consideration to the protection of intellectual property associated with this work and that there are no impediments to publication, including the timing of publication, with respect to intellectual property. In so doing we confirm that we have followed the regulations of our institutions concerning intellectual property.

We understand that the Corresponding Author is the sole contact for the Editorial process (including Editorial Manager and direct communications with the office). He is responsible for communicating with the other authors about progress, submissions of revisions and final approval of proofs. We confirm that we have provided a current, correct email address which is accessible by the Corresponding Author and which has been configured to accept email from plesibana@gmail.com.

The researchers adhered to the ethical guidelines the Turfloop Research Ethics Committee (Ethical No: TREC/290/2017: PG). All informants completed the Standard University of Limpopo Consent form.

Signatures

Lesibana P. Maema Martin J. Potgieter Amidou Samie


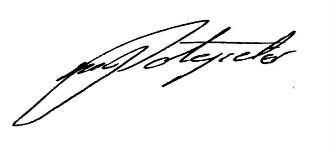

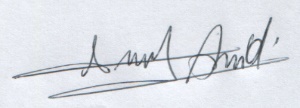
 20 March 2019 Date Date
